# Modelling the size, cost and health impacts of universal basic income: What can be done in advance of a trial?

**DOI:** 10.1007/s10742-021-00246-8

**Published:** 2021-04-11

**Authors:** Matthew Thomas Johnson, Elliott Aidan Johnson, Laura Webber, Rocco Friebel, Howard Robert Reed, Stewart Lansley, John Wildman

**Affiliations:** 1 Politics, Philosophy and Religion, County South, Lancaster University, Lancaster, LA1 4YL UK; 2 HealthLumen, London, UK; 3 Department of Health Policy, The London School of Economics and Political Science, Houghton St, London, WC2A 2AE UK; 4 Landman Economics, Colchester, UK; 5 University of Bristol, London, UK; 6 Health Economics, Population Health Sciences Institute, Faculty of Medical Sciences, Newcastle University, Newcastle upon Tyne, UK

**Keywords:** Universal Basic Income, Social determinants, Modelling, Health impact, Tax

## Abstract

Opposition to Universal Basic Income (UBI) is encapsulated by Martinelli’s claim that ‘an affordable basic income would be inadequate, and an adequate basic income would be unaffordable’. In this article, we present a model of health impact that transforms that assumption. We argue that UBI can affect higher level social determinants of health down to individual determinants of health and on to improvements in public health that lead to a number of economic returns on investment. Given that no trial has been designed and deployed with that impact in mind, we present a methodological framework for assessing prospective costs and returns on investment through modelling to make the case for that trial. We begin by outlining the pathways to health in our model of change in order to present criteria for establishing the size of transfer capable of promoting health. We then consider approaches to calculating cost in a UK context to estimate budgetary burdens that need to be met by the state. Next, we suggest means of modelling the prospective impact of UBI on health before asserting means of costing that impact, using a microsimulation approach. We then outline a set of fiscal options for funding any shortfall in returns. Finally, we suggest that fiscal strategy can be designed specifically with health impact in mind by modelling the impact of reform on health and feeding that data cyclically back into tax transfer module of the microsimulation.

## Introduction

The COVID-19 pandemic has seen increased interest in Universal Basic Income (UBI) as a means of mitigating poverty and insecurity associated with its socio-economic fall-out (see for example Moran 2020). We have asserted that such mitigation means that, in theory, UBI can serve as an upstream public health measure by addressing socio-economic determinants of health ([Redacted]). The precise impact can only be addressed via a trial designed and evaluated specifically with health impact in mind. However, opposition to a trial of UBI of sufficient size and scale has often been grounded in an intuition summarised by Martinelli (2017, 43) that ‘an affordable basic income would be inadequate, and an adequate basic income would be unaffordable’. This assessment neglects the potential economic returns of a successful upstream intervention aimed at promoting health. Indeed, no proposal for UBI has, as yet, designed the size of cash transfer, quantified its prospective benefit or evaluated cost with that impact in mind.

**Fig. 1:**
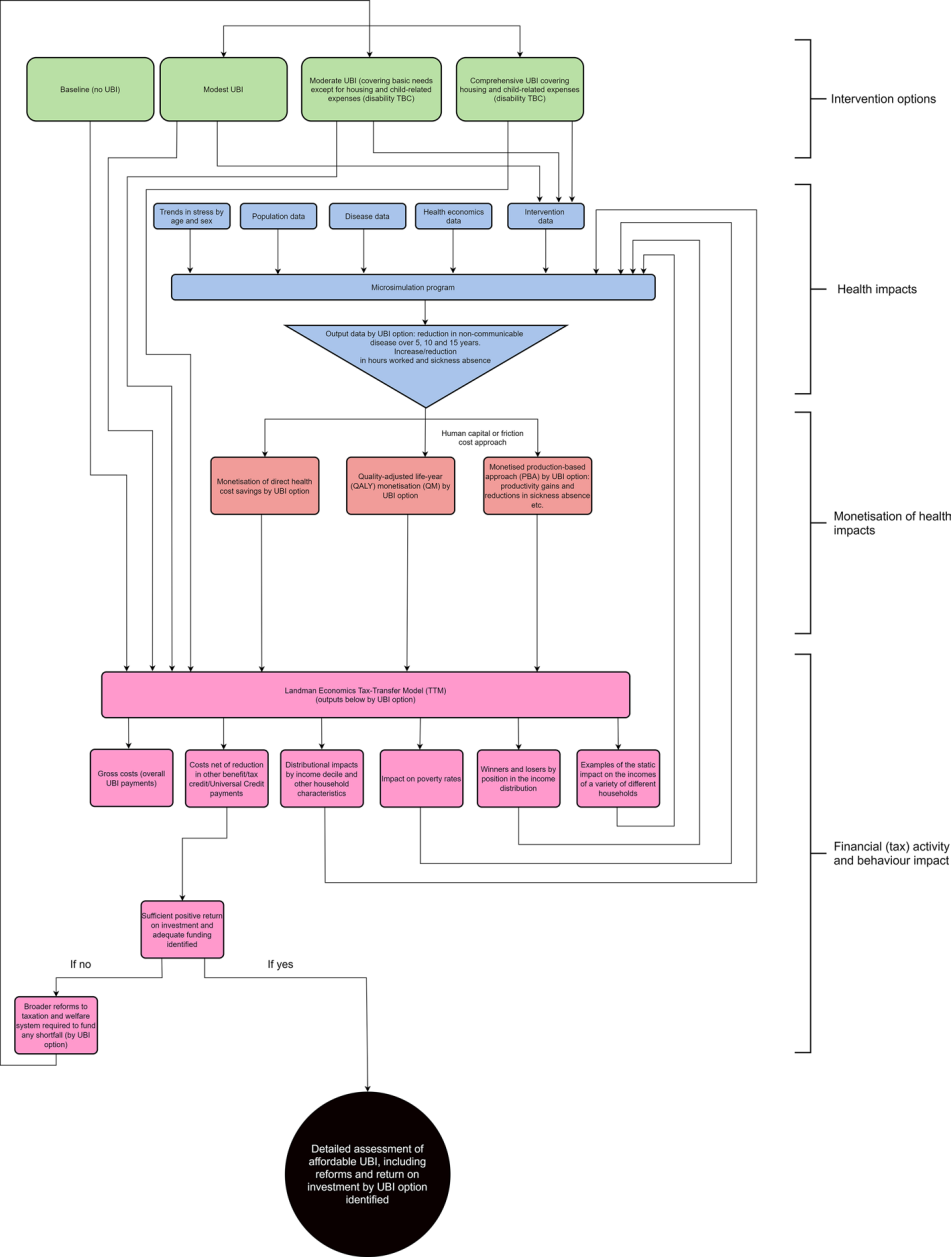
Process of establishing viability of UBI via health impact

In this article, we present a methodological framework (see Fig. 1 for assessing prospective costs and returns on investment in advance of a trial in order to make the case for the trial itself. We begin by outlining the pathways to health in our model of change in order to present criteria for establishing the size of transfer capable of producing measurable positive health impacts. We then consider approaches to calculating cost in a UK context to estimate budgetary burdens that need to be met by the state. Next, we suggest means of modelling the prospective impact of UBI on health before asserting means of costing that impact. We then present a strategy for building that costed impact into a microsimulation of economic impact before outlining a set of fiscal options for funding any shortfall in returns. Importantly, we suggest that fiscal strategy can be designed specifically with health impact in mind by modelling it and feeding that data cyclically back into tax transfer microsimulation. Setting out the methodological framework by which to examine affordability constitutes a significant step forward for integration of the health case into evaluation of UBI and provides a platform for research to make the argument for a trial. We set aside, here, other economic impacts such as crime reduction, additional consumer spending, increased skill development in the workforce, potential pay increases and inflation, but note their importance.

## Pathways to health

Johnson, Johnson, Nettle and Pickett (2020) have presented three primary pathways to health through the introduction of UBI as an upstream intervention. Each stems from effects on higher level social determinants and feeds down through individual determinants of health to direct health impacts and on to public health impact. First, where UBI constitutes an increase in income, it can reduce poverty. Reducing poverty improves food sufficiency and enhances the quality by which basic needs are satisfied (Johnson, Degerman and Geyer 2019). This pathway may be indicated by data arising from cash transfer programmes, with both the minimum income guarantee in Gary Indiana and the Alaska Permanent Dividend Fund having a positive impact on birth weight, for example (Kehrer and Wolin, 1979; Chung, et al. 2016). Second, where UBI enhances predictability and security of income by acting as a universal and equal entitlement, it can reduce stress associated with exposure to threat (Johnson and Johnson, 2019). While stress is generally treated as a psychological phenomenon (Kangas, et al. 2019, 25), long-term exposure under threatening social circumstances (e.g., workplace and domestic bullying and abuse) contributes to a wide range of stress-related conditions. Having an adequate and predictable, secure income mitigates inequalities that provide the social basis for stress. UBI may, therefore, achieve the changes that Thoits (2010, S47) calls for in upstream interventions: a reduction in ‘health inequalities and the structural conditions that put people “at risk of risks”’, such as ‘discrimination, poverty, residential segregation, inadequate schools, unemployment’ (Thoits, 2010, S47). According to Kangas, et al. (2019, 25), ‘the predictability of the basic income is thought to reduce the level of stress due to less bureaucracy and more certain flow of income’. Third, and as a result of both poverty reduction and security enhancement, UBI can alter people’s behaviour. Individuals facing scarcity or unpredictability in their lives may invest less in behaviours that promote their long-term health and well-being (Pepper and Nettle 2017). Where UBI fosters predictability, it can promote longer-term thinking that contributes to health and well-being (Johnson, Degerman & Geyer 2019). This may explain a decrease in health-reducing behaviour among recipients of Tribal Cash Transfers (Costello et al. 2010).

Each of these pathways require the size of UBI to be significant. Indeed, concern for a safety-net indicates that UBI needs to satisfy people’s basic needs in order to be impactful. How expensive would this be for the public purse?

## Minimum income standards and budget implications

The pathways to health impact depend upon satisfying individuals’ basic needs. If individuals are able independently and adequately to satisfy those needs, they are not subject to extremes of poverty or to threatening inequalities that place them at risk of destitution. The theoretical model indicates, that while improvements in income may improve some health outcomes, income that falls below the level needed to satisfy basic needs means that individuals will not be able to achieve others. While there have been many different microsimulation calculations for a welfare framework that achieves a basic minimum with reduced conditionality (see, for example, Lansley and Reed 2019), concern for stress associated with conditionality provides a *prima facie* health-based rationale for streamlining systems around a single ‘adequate’ payment (Johnson and Johnson, 2019).

In the UK, perhaps the most prominent formula is the Minimum Income Standard (MIS), which was developed by the Centre for Research in Social Policy (CRSP). This is designed to show ‘what UK households need today in order to have a decent living standard, considered the minimum by the general public’ (Hirsch 2019, 4). The MIS for 2019 for a single person with no children was £313.68 per week (net of direct taxes, such as income tax and national insurance, but gross of council tax) (Hirsch 2019, 7). If the aim of UBI is to satisfy basic needs while replacing all forms of means-tested support, including housing (though without accounting for local differences in cost), UBI payments would equate to £1,359 per month per adult or £16,311 per year. Most UBI schemes are designed with payment to adult citizens in mind. However, establishing that figure in the UK is functionally difficult in the absence of more precise record keeping. As such, provisionally, the closest figure is the total number of residents, suggesting the number of recipients to be 52.4 million in the UK in 2018 (Office for National Statistics 2019). This excludes individuals living overseas who are at present entitled to state pensions and ignores conditions on residence, such as temporary leave to remain. The provisional cost for a full MIS payment would be £854.7bn per year.

This MIS figure holds that single adults with no children need £92.49 per week for rent, close to the average housing benefit payment of approximately £101 (Department for Work and Pensions 2019a, Table 1a). Removing this brings the payment to individuals down to £221.19 per week, £958 per month, £11,502 per year and cost to the state to £602.7bn. ‬Removing council tax from MIS brings the payment down to £203.90 per week, £884 per month, £10,603 per year and cost to the state to £555.6bn.

A second potential approach to calculating size of payment deploys relative poverty. In 2017/18, 60% of the median weekly income for single adults of working age without children was £204 per week (McGuinness, Booth and Francis-Devine 2019, 43), £884 per month, £10,608 per year or £555.9bn for the adult population. Although the lower end MIS figure is similar, this relative poverty calculation includes all income, including state support, after tax and statutory deductions (Department for Work and Pensions, 2019b, 14). While the similarity in figure grants credence to the notion of a minimum standard, the caveat above and the fact that calculations of relative poverty not relating directly to the cost of satisfying basic needs indicates that there are risks of such figures underestimating costs. As such, there are good reasons to operate with three MIS figures representing a range from minimum to maximum annual payments and budgets: (i) £10,603 per person, £556.6bn budget; (ii) £11,502 per person, £602.7bn budget; (iii) £16,311 per person, £854.7bn budget.

This leaves open a series of additional budgetary questions. First, how should those MIS payments that exclude housing costs be supplemented? Housing Benefit is forecast to be awarded to 4.5 m people and to cost an average of approximately £101 per week/£5,231 per year per person and £23.4bn overall in 2019/20, (Department for Work and Pensions 2019a, Table 1a). In November 2019, the average housing benefit (excluding Universal Credit Housing Element figures) ranged from £79 in Yorkshire and The Humber to £152 in London (Department for Work and Pensions 2020). Extending the average housing benefit payment as part of UBI to the whole adult population would cost £274.1bn per year. Given that council tax and rent vary substantially depending on location–and as household structure is not uniform–the pared back MIS figure may reflect a nationally applicable individual minimum. This leaves policymakers with an open question about how to trade off cost for administrative simplicity.

Second, what support should there be for children? Options include: administratively burdensome, but cheaper, means-tested benefits, such as Personal Tax Credits (now becoming part of Universal Credit), Tax Free Childcare and Universal Credit child payments; a less administratively burdensome, but more expensive, under-18 s UBI (see Standing 2019, 49; Lansley & Reed 2019), which could be based on an expanded Child Benefit if the High Income Child Benefit Charge (HICBC) were abolished, and replacement of benefits with expanded public services, e.g. public childcare, which has not been costed, though other Universal Basic Services have (see Portes, Reed & Percy 2017). Leaving current systems in place would add the following: £11.6bn (in 2019/20) for Child Benefit (and similar benefits), which currently pays a first-born or only child £20.70 and additional children £13.70 per week;–£950 m for Tax Free Childcare (in 2023/24 as it gradually replaces Childcare Vouchers); and £24.8bn on Child and Working Tax Credits (Department for Work and Pensions 2019a, Non-DWP welfare).

Third, how should disabled people be supported? Research by Scope, the disability equality charity, (John, Thomas and Touchet 2019) indicates that disabled adults face additional costs of £583 per month even after taking benefits into account to have the same standard of living as their non-disabled peers. Means of meeting those costs through Personal Independence Payment (PIP), Disability Living Allowance (DLA) and Attendance Allowance were forecast to cost the Exchequer £26.3bn in 2019/2020 (Department for Work and Pensions 2019a, Table 1a). The potential loss of targeted financial assistance was one of the key concerns expressed by Disabled People Against Cuts toward introduction of UBI (DPAC) (2019, 9). It is entirely possible to introduce UBI without touching needs-based benefits (Lansley and Reed 2019, 10; Stirling and Arnold 2019) or to include a supplement within a UBI transfer (Standing 2019, 36). However, beyond arbitrary decision making (see Pybus, et al. 2019), there is substantive evidence that assessment in provision of conditional support harms the health of disabled people by disincentivising activity or incentivising inactivity and drug use to indicate ill-health (see Johnson and Spring 2018; Activity Alliance and IFF Research 2020; Johnson, Degerman and Geyer 2019). Retaining conditionality requires substantial, and ultimately inclusive, reform of assessment in ways that mitigate the effect of assessment on health. This is an extremely challenging task, given the central importance of health to this justification, the difficulty of assessing without stress-inducing intrusion, and the cost of administering conditional systems, with assessments estimated to cost more money than they saved identifying fraud between 2016 and 2019 (Comptroller and Auditor General 2016).

‬‬Fourth, how should residency and citizenship be evaluated with regard to entitlement? The three budgetary calculations do not include costs for the 1.17 m (Thurley and McInnes 2020, 12) overseas pensioners. A decision would be needed on whether their entitlement to a legacy pension would translate into entitlement to UBI. Wider examination of eligibility conditions based on residence and nationality is also required. Some 6.2 m UK residents held a non-UK nationality in 2019 (Sturge 2020, 3), though the number with indefinite leave to remain is unclear. Also, the United Nations (2019, Table 1) estimates that there are approximately 3.8 m people born in the UK who now reside in other countries.

## Reassessing martinelli

Martinelli (2017) provides a cost–benefit assessment of five UBI schemes. These ranged from a low-level transfer of £42.19 per week to all adults and children–a cost to the Exchequer of £140bn–to a high-level transfer of £115.29 for working age adults, £197.79 for pensioners (65 + for men and 62 + for women) and £109.20 to children and young people aged 17 and under–at a national cost of £427bn. However, as these transfers are significantly lower in cost than those suggested above, their impact on health may be less impactful.

As with many analyses of prospective UBI schemes, Martinelli places significant weight on pre-COVID-19 assumptions regarding political feasibility. Indeed, it is political feasibility, that leads Martinelli to discount the possibility of introducing ‘a full basic income–with all the benefits that might come with it’ (2017, 74), in part on the basis of the increased cost beyond current welfare budgets. On the other hand, Martinelli accepts that the introduction of an ‘interim’ option at a much smaller level of transfer is ‘not without danger’, as it is ‘not clear that partial models will give rise to full complement of advantages on which basic income is sold (2017, 74). This leads him to conclude that a partial system.

may represent the worst of both worlds: UBI may be unable to ‘piggyback’ on existing systems and institutions, requiring brand new ones operating alongside those that already exist. In such a situation, UBI could represent greater rather than reduced administrative complexity and cost. (Martinelli 2017, 74)

There are three crucial reasons for rejecting Martinelli’s conclusions. First, perceptions of public spending have been transformed by the pandemic (Nettle, Johnson, Johnson & Saxe 2021), in which greatly enhanced public spending has become the norm. This extends even to governments, including in the UK, that have traditionally been committed to fiscal conservatism. Second, the microsimulations that have been undertaken, referenced by Martinelli (2017, 49), are static models. As such, the benefits and cost-savings side of the calculation fail to account for the changes in behaviour and the cyclical benefits that may result from the introduction of UBI. Indeed, modelling that has been conducted dynamically, such as the Fraser of Allander Institute model, focuses solely on macroeconomic impacts and specifically excludes health impacts from the analysis (Fraser of Allander Institute 2020, 45). Third, the evidence examined by Martinelli and others is derived from transfer programmes directed at low income or particular ethnic or regional groups. Provision of a secure, adequate income is likely to provide health benefits, including via reduced physiological stress, for almost all in society, not solely those targeted in small-scale trials on which Martinelli’s claims are built. Fourth, ‘inadequate’ schemes, such as those assessed by Martinelli, are also least likely to provide an economic return on investment. Evidence from studies such as Whitehall II indicates that certain health benefits can only be accrued from provision of secure, stable income of a sufficient level to satisfy basic needs independently of employment and unreasonable job demands.

These four responses to Martinelli provide grounds for consideration of an ‘adequate’ payment. As Fig. 1 indicates, there are several considerations presented by the health case that shift concern for cost-neutrality toward the impact of improved health on the economy. In order to understand that impact and to make the case for UBI, we need to be able to model the prospective impact of the identified pathways in advance of any trial. We will do so with regard to three different schemes: a starter scheme to supplement existing benefits, an intermediate scheme that replaces some benefits and keeps others (housing benefit, etc. and a full MIS scheme that replaces all benefits, as outlined above.

## Modelling pathways

While well designed, randomised controlled trials are often viewed as a gold standard, they are constrained by cost and the electoral cycle, with programmes given a short window of often 2–3 years in which to demonstrate impact. This is particularly problematic for a health-based trial of UBI, since some prospective impacts are cascading and, in the case of disease avoided, occur beyond the trial cycle. Where trials are conducted, they are generally introduced to a subset of a given population, meaning that it is unclear how that intervention might play out when scaled to an entire population. Therefore, mathematical modelling is a crucial complement to trials by enabling the long-term health and economic impact of a given intervention to be quantified before real world implementation. Simulation models can also be used to complement trials, by using that trial data to quantify health impacts over 5, 10 and 20 years to account for different aetiological and political periods, where it is not ethically or economically viable to run the trial over a long timeframe. Indeed, given that the aetiological period of behaviours, such as smoking, extends into decades (see Doll, et al. 2004), and given that one prospective impact of UBI is to affect behaviour, no ‘trial’ could record such impacts as part of the evidence gathering phase of policy development. As such, modelling is a crucial part of the decision-making process, providing evidence for policy in the cases of the UK Government’s response to COVID-19 (Verity, et al. 2020), the UK Soft Drinks Industry Levy (Briggs, et al. 2013) and the Scottish Government’s minimum unit pricing of alcohol (Katikireddi, Hilton and Bond 2016).

Modelling, in this instance, may provide significant evidence of impact in advance of a trial. For example, non-communicable diseases (NCDs), such as heart disease, diabetes, respiratory disease and dementia account for 71% of all deaths, globally (WHO 2020). NCDs shorten lives, cost governments an increasing amount of money and will push millions of people below the poverty line in the coming decades. Therefore, preventing and reducing NCDs is a priority both in the UK and globally. NCDs take many years to unfold and are often preceded by stress-related metabolic syndrome (hypertension, high cholesterol and reduced responsiveness to insulin) (Fricchione 2018). With regard to our model of impact, higher level social determinants may also indirectly cause NCDs by promoting NCD risk factors, such as increased frequency of smoking, substance abuse, increased alcohol consumption and disordered eating (Ghosh and Verma, 2018; Linsky, Straus and Colby 1985; Welch, Doll and Fairbairn 1997). This indicates that positively affecting individual determinants of health, such as stress, via an intervention like UBI offers significant potential for impact.

To estimate that impact, pre-trial modelling is necessary. This requires a combination of data from earlier, similar interventions alongside expert assumptions to assess different scenarios of UBI success on health and related economic outcomes. Epidemiological risk factors for direct health impacts (see Fig. 1) can be sourced from national health surveys such as the Whitehall II study, Health Survey for England (NHS Digital 2020), Annual Population Survey (Office for National Statistics 2012) and English Longitudinal Study of Ageing (2020). Disease data can be drawn from literature, and dynamic population data can be sourced from the Office for National Statistics (2020a, b). In the absence of reliable estimates of UBI’s health impacts from an existing trial, it is feasible to derive proxy data from conditional cash transfer programmes (Huntington 2010), which could inform prospective modelling. Other possible sources of information could come from policy effects related to social welfare programmes, including minimum wage policies (Leigh and Du 2018), introduction or removal of child benefit payments (Milligan & Stabile 2009) or impacts of pension schemes on health (Hultin, Lindholm & Moller 2012). While qualitative assumptions are not ideal, when coupled with existing trial data they provide the best estimation of long-term health impact of UBI under the different possible schemes. Sensitivity analysis, whereby individual parameters are adjusted to quantify their impact on the overall effect, is important for parameterising trials for maximum effect.

Microsimulation models (such as that illustrated in blue in Fig. 1) can be adapted to include the individual level determinants as risk factors for later NCDs. Such models simulate a virtual population of 20 + million people based on known population statistics, births and deaths. Each year the number of new stress- or lifestyle-related diseases can be quantified based on age, sex, socio-economic distribution in the ageing population and trends in stress over time. Each individual is allocated a level of each of the individual determinants on the basis of observed population distributions. For example, measures of stress (e.g., cortisol measurements from the Whitehall Study) give each individual an annual probability of contracting, surviving or dying from a stress-related condition (e.g., cardiovascular disease). This permits comparison of a baseline (trends in stress and related disease continue without intervention) against a UBI scenario, in which stress is reduced across the population and/or in specific socio-economic groups in proportion to the impact of the payment on poverty, unpredictability and behaviour. The model can also quantify the indirect effects of UBI on NCDs via its impact on the other individual determinants of health, such as the multiple interacting risk factors of behaviour relating to diet, smoking and alcohol consumption, that tends to cluster in lower socio-economic groups (Birch, et al., 2019). Combined relative risks are required to account for the interacting and/or overlapping risks of these health behaviours, to ensure the model does not double count nor underestimate outcomes. For instance, Hart, et al. (2010) showed that the relative risk (RR) for body mass index on liver disease mortality was 1.29 (0.60–2.80), for alcohol on liver disease it was 3.66 (1.74–7.71) but the excess risk due to the interaction between BMI and alcohol on liver disease was more than double, at 9.53 (4.98–18.2) highlighting a ‘supra-additive’ interaction. Such epidemiological phenomena need to be accounted for in the modelling. It may be possible to estimate some direct health impacts in different groups from analysis of data on impact of other measured economic interventions, such as in Gibson, Hearty and Craig’s (2020) review of programmes that resemble UBI as well as proxy data from conditional cash transfer programmes (Huntington 2010).

However, the data on which this modelling depends is incomplete, with no trial having been designed and measured for health impact and Kontis, et al. (2015, e747) claiming that stress is poorly measured in other interventions. As such, significant epidemiological reviews are required in order to ensure that the estimate is indicative of impact. This, though, simply emphasises the need for a health-based trial to produce the data to ‘feed’ modelling on the effect on individual determinants of health before and after UBI or between the control and experimental groups. This data collection is required to make robust predictions about where UBI has impact, over what time period, and in which groups. Even so, the modelling derived from review of the epidemiological data can provide the basis for an informed estimate of impact. How, though, can that impact be monetised in order to establish a prospective return on investment?

## Costing health impacts and quantifying economic return on investment

Given the pressure on health budgets as a result of a sustained period of austerity (see Local Government Association 2019) and the COVID-19 induced recession, it is essential that any substantive intervention be assessed on its returns. With the value of health systems at the forefront of the public’s mind and the UK Government committed to a ‘prevention agenda’ (Department of Health and Social Care 2018) intended to shift understanding of the NHS away from a ‘National Hospital Service’ (see Department of Health and Social Care and Hancock 2018), a monetised health impact is particularly salient. In this context, understanding health impact is essential precisely because of its substantive monetary value. For example, stress as one individual determinant of health was found to be responsible for ‘37% of all work-related ill health cases in 2015/16, and 45% of all working days lost due to ill health’ in Great Britain (Health and Safety Executive 2016, 2). Among others, it has also been linked to long-term health conditions such as heart disease, stroke, cancer, type 2 diabetes, arthritis and depression (Department of Health 2012, 5), which are responsible for 70% of NHS England expenditure, representing 50% of all GP appointments, 64% of outpatient appointments and 70% of all inpatient bed days (Department of Health 2012, 3). Any intervention capable of reducing stress, such as UBI, offers significant potential for return on investment.

To support the decision-making process, all direct and indirect costs as well as all benefits resulting from UBI have to be monetised. In line with the WHO Guide to Cost-Effectiveness Analysis, this includes an evaluation of costs and outcomes from a societal perspective, which will account for the opportunity costs in the UBI funding decision. It then allows for a cost–benefit analysis (CBA) that can act as a decision-making tool for policymakers (Brent 2013). The CBA, which is rooted in welfare economics, quantifies the level of return for a given level of investment (Park, Jit & Wu 2018) and has been commonly applied to support decision-making processes, particularly for public health interventions (Owen, Pennington, Fisher & Jeong 2018). It is endorsed by the National Institute for Health and Care Excellence (NICE) with robustness to secure support from health commissioners in the UK, and internationally.

For the investigation into the impact of UBI, the outcome for a CBA would be the total cost associated with the UBI scheme, which can be defined as the total transfer amount in Pound Sterling for the entire target population and the administrative costs in distributing the transfer to the recipients. Point estimates of costs can be used to produce a central cost scenario, with upper and lower confidence intervals providing statistical bounds for robustness analysis.

While data provided by trials often present short-term costs and benefits, CBA considers longer term impacts, usually over a 10-year time period. Though, longer term horizons could be considered, especially when impacts of UBI materialise further in the future. However, this will likely incorporate higher levels of uncertainty, with subsequent impacts on the robustness of CBA estimations.

The data entered into the CBA model in this instance would be derived from the microsimulation modelling above. The direct benefits of UBI on health include all medical costs/health service costs avoided due to reduced levels of disease (e.g., NCDs triggered through stress). Monetary values for direct benefits can be derived from NHS reference cost data provided by NHS England and NHS Improvement (2020), comprising the average unit cost of providing defined services to NHS patients and costs based on specific interactions between patients and providers related to their healthcare activity. NICE recommends using a discount rate of 1.5% per year for public health interventions, alongside a sensitivity analysis using the Treasury Green Book discount rate of 3.5% (NICE, 2012).

A combination of two approaches could be used for the monetisation of indirect benefits: the quality-adjusted life-year (QALY) monetisation (QM) approach and the production-based approach. First, under the QM approach, individual Willingness to Pay (WTP) in monetary terms for an additional QALY gained can be estimated, using existing evidence on the WTP for incremental changes in QALYs from a study by Shiroiwa, et al. (2009). In accordance with cost-effectiveness thresholds used by NICE, a monetary value of between £20,000 and £30,000 can be attached per discounted QALY.

Second, the production-based approach (PBA) assesses indirect UBI benefits related to productivity loss averted because of decreased levels of mortality and morbidity. This approach regards health as human capital, rather than as intrinsic value (see Grossman 1972). A key assumption of the PBA is that sick or deceased workers are irreplaceable. Therefore, productivity loss due to sickness remains the same across the treatment period (*i.e.*, until previous health state is resumed), while productivity loss due to premature death can be estimated using standard retirement age as a relevant cut-off (*i.e.*, 65 years). Productivity loss can then be estimated as the cumulative income loss for the period of sickness (workplace absenteeism), and income losses due to premature death can be calculated using data on population-stratified employment rates and mean incomes from the Office for National Statistics (HM Revenue & Customs 2020b; ONS 2018a; ONS 2020b).

To the extent that positive health feedback effects from a UBI scheme can potentially improve the rate of productivity growth overall, this can be modelled by starting with the Office for Budget Responsibility’s long run productivity growth assumptions from its Fiscal Sustainability Report (OBR, 2020) and amending them as required. For example, the results from the positive health impacts of the UBI package might suggest an increase in average wages in the economy, or changes in the employment rate. Changes in these key macroeconomic parameters can be fed back into the tax-transfer model to calculate a new set of results for tax receipts and welfare spending–and hence a new set of fiscal impacts of the UBI–into the future. The Tax Transfer Model (TTM) has been used in this way in previous research to model the future impact of changes in employment rates and wage and productivity growth on the public finances and other key indicators such as poverty rates and the degree of inequality in the UK (see for example Reed and Portes 2014, Harrop and Reed 2015).

There are several alternative approaches to the human capital consideration with potential impacts on the overall estimate of UBI benefits, including the friction cost approach. Unlike the human capital approach, it considers the employer perspective and accounts for market fluctuations in employment by substituting sick or deceased workers with those in a pool of unemployed. As a result, productivity losses are assumed to be much smaller, since they appear only in the friction period. Both approaches only consider paid employment, but it is also important to value the economic contribution of unpaid workers. This includes those who provide childcare, adult and social care, household services, voluntary services and lifts the burden particularly for sectors funded through public accounts. While the contribution of unpaid work is difficult to disentangle, according to the Office for National Statistics (ONS 2018a, b) it was estimated at £1.24 trillion, or equivalent to 63.1% of Gross Domestic Product for financial year 2015–16. As outlined by Park, et al. (2018), the UK Government has been recognising the value of unpaid work and it would be sensible to attach a monetary value equivalent to the average UK wage or median household wage. Finally, a standard discount rate of 3.5% per year would be applied to future deaths averted by taking the implementation of UBI as reference case. The same discount rate would apply for the period to year of death for losses in productivity and QALYs because of premature mortality.

These monetised data can then be entered into a TTM of the tax-benefit system. The TTM uses data from the Family Resources Survey (FRS) to analyse the impact of direct taxes, benefits, tax credits and Universal Credit, and data from the Living Costs and Food Survey (LCF) to model the impact of indirect taxes. The information in the FRS allows payments of direct taxes and receipts of benefits, tax credits and/or Universal Credit to be modelled with a reasonable degree of precision for each household in the FRS using either the current tax-benefit system in place at the moment, or an alternative system of the user’s choice. The TTM can be used to estimate the immediate, static ‘first-round’ impact of each of the three UBI options on individual and household net incomes in the UK using data from the Family Resources Survey micro dataset. The outputs from the analysis would include:

- Gross costs (overall UBI payments).
- Costs net of reduction in other benefit/tax credit/Universal Credit payments.
- Distributional impacts by income decile and other household characteristics.
- Impact on poverty rates.
- Winners and losers by position in the income distribution.
- Examples of the static impact on the incomes of a variety of different households.

The modelling can then be repeated to include the dynamic impacts at 5 and 10 years to reflect data on savings from lower sickness levels, lower levels of spending on national health, and the wider employment impact. These ‘second’ and ‘third’ round impacts can be factored back into the modelling, altering the modelled fiscal impacts of each of the UBI options. Additional data on the effect of cash transfers on labour market behaviour, including early retirement, can be introduced to calculate impacts on tax revenues. A previous example of fiscal savings calculations of this kind is Reed (2010) who considers the impact of increased tobacco taxation (and consequently, reduced smoking prevalence) on the public finances via a number of mechanisms, including: reduced NHS costs; increased employment due to lower levels of early retirement due to ill health, lower absenteeism, and higher productivity at work.

Concern for the intrinsic, as well as instrumental, value of health can then be used to consider strategies for funding shortfalls in budgets. How, though, can these schemes be funded?

## Reform of government spending

It is important, not just that the potential for economic impact be projected, but that thoroughgoing examination of fiscal policy be undertaken. To achieve cost-neutrality for an ‘adequate’ UBI, significant reform would still be necessary. Financing MIS-based UBI schemes would require substantial changes to the tax system, for individuals and corporations. Replacing pensions (£164.6bn), central and local government welfare (£113.8bn) (Chantrill 2020) and the tax-free personal allowance (£111.2bn) (Stirling and Arnold 2019) would generate £389.6bn, annually. This would allow for a UBI of £7,435. To get to £10,603, as the minimum MIS-based figure, another £165.8bn, independent of additional funding for supplementary payments to children and disabled people, would be needed beyond the current spent on personal financial support to achieve cost neutrality.

Given that the up-front cost is significant, one option might be to meet the early years costs from the issue of long-term Government 10–20 year bonds. At today’s near zero interest rates, the annual cost of such bonds is close to zero. This would reflect the fact that there would be net economic gains accruing over time, which would be sufficient to pay the capital when due. This would enable gains to be capitalised from the scheme’s introduction. Lansley and Reed (2019) have already suggested other options, including by: raising the rate of corporation tax (a rise of 1p raises £2.6–2.8bn) (KAI Indirect Taxes, Customs and Coordination 2018); reducing the number and cost of tax reliefs beyond personal tax-free allowance, which the Office of Tax Simplification has identified as numbering 1,156 (Sinfield 2018) and which cost the Exchequer around £400bn annually in total, including £41bn per annum in pension tax relief, £34bn in corporation tax and capital gains tax for business assets (Miller 2018; House of Commons Treasury Committee 2018) and ‘Entrepreneur’s relief’ for company owner-managers, which cost £2.7bn per annum and has been poorly targeted (Corlett 2018); a phased reduction in financial support to home owners and private landlords, which has an impact on house prices and disproportionately benefits property developers, which costs £8bn a year (Wilcox & Williams 2018); extending NICs to those over 65, a change advocated by the Inter-Generational Foundation as a way of improving inter-generational fairness (Office for National Statistics 2017); raising the revenue yield from the new digital services tax on big technology companies, which raises £400 m per annum at present (Hern 2018); reversing the freeze in diesel and petrol excise duties since 2010, which costs £9bn per annum (Lansley & Reed 2019, 23); increasing rates on existing eco-taxes (Gov.uk 2020), and introducing new levies on certain forms of privately owned wealth, which currently stands at some £14.6tr, some seven times the size of national income (Office for National Statistics 2019). These options stand aside from revision of marginal tax rates, introduction of Land Value Tax or Financial Transaction Taxes, each of which would produce significant yields.

Vitally, modelling permits consideration of the impact of fiscal strategies on health and wellbeing. The strategies, and their impact on higher level social determinants of health can then be fed back into health microsimulation modelling to establish health impact, which can be monetised and then fed back into the economic microsimulation modelling to determine a final framework for achieving cost neutrality. This cyclical dynamic modelling has not previously been conducted and has the potential to deliver a much clearer assessment of the viability of different UBI schemes.

Sensitivity analysis of the results can be conducted in two ways. Firstly, where inputs to the TTM (such as wage levels, employment rates, the distribution of population health parameters, etc.) are based on a modelling approach which produces standard errors and confidence intervals (for example, regression-based modelling), the confidence intervals can be used to provide upper and lower bound estimates. Secondly, specific scenarios can be designed to test the impact of potential future changes to the economy–for example, increased automation in the labour market. The second approach is particularly important for simulating effects further than 10–15 years into the future, where the fundamental parameters of the economy are likely to be subject to significant–and perhaps transformational–technological change.

## Conclusion

Establishing the economic impact of upstream public health interventions is an essential contribution to debate on UBI. The only means of establishing that impact precisely is via a large-scale trial. However, as we suggest above, there are plausible means of developing theoretical schemes, modelling their impact and developing a funding framework in advance of a trial. This is crucial, since discussion of the policy is circumscribed by concerns for cost that Martinelli has expressed so forcefully. The model of change presented in Fig. 1 has the capacity to transform that debate, building on the work that has already been done by Lansley and Reed (2019) and others to suggest the theoretical possibility both that a larger, unconditional UBI has the capacity to deliver qualitatively distinct health impacts via stress reduction and behavioural change than smaller payments, and that those benefits have the potential to produce economic returns that bring the policy closer to cost neutrality. Indeed, concern for health can be embedded in assessment of fiscal strategies to fill any gap by feeding the economic impacts of reform into simulation and costing of further health impacts and, cyclically, back into economic microsimulation. This is important, insofar as it permits modelling of the three different schemes and three different levels of attendant reform that we develop. The theoretical account above suggests that a realignment of state support from corporations to individuals could deliver a budget required to achieve cost-neutrality for an adequate UBI. This is before potentially substantial health and social care cost savings from improvement in health and provision of caring support from family members is considered. The most important contribution of this article is establishing, in Fig. 1, a methodological framework for the substantive research required to complete such modelling. This constitutes a significant step forward in efforts to evaluate substantively a policy invoked or rejected on the basis of disparate evidence.
